# Microarray phosphatome profiling of breast cancer patients unveils a complex phosphatase regulatory role of the MAPK and PI3K pathways in estrogen receptor-negative breast cancers

**DOI:** 10.3892/ijo.2014.2648

**Published:** 2014-09-09

**Authors:** RAMON G. MANZANO, ELENA M. MARTINEZ-NAVARRO, JERONIMO FORTEZA, ANTONIO BRUGAROLAS

**Affiliations:** 1 Molecular Genetics and Genomics Laboratory, Plataforma de Oncologia, Hospital Quiron Torrevieja, 03184 Torrevieja, Alicante, Spain; 2 Department of Pathology, Plataforma de Oncologia, Hospital Quiron Torrevieja, 03184 Torrevieja, Alicante, Spain; 3 Department of Medical Oncology, Plataforma de Oncologia, Hospital Quiron Torrevieja, 03184 Torrevieja, Alicante, Spain; 4 Complejo Hospitalario de la Universidad de Santiago de Compostela, Santiago de Compostela, La Coruña, Spain

**Keywords:** microarray, breast cancer, phosphatome, phosphatase, DUSP4, DUSP6, MAPK, PI3K, profiling

## Abstract

Phosphatases are proteins with the ability to dephosphorylate different substrates and are involved in critical cellular processes such as proliferation, tumor suppression, motility and survival. Little is known about their role in the different breast cancer (BC) phenotypes. We carried out micro-array phosphatome profiling in 41 estrogen receptor-negative (ER^−^) BC patients, as determined by immunohistochemistry (IHC), containing both ERBB2^+^ and ERBB2^−^ in order to characterize the differences between these two groups. We characterized and confirmed the distinct phosphatome of the two main ER^−^ BC subgroups (in two independent microarrays series) and that of ER^+^ BC (in three large independent series). Our findings point to the importance of the MAPK and PI3K pathways in ER^−^ BCs as some of the most differentially expressed phosphatases (like DUSP4 and DUSP6) sharing ERK as substrate, or regulating the PI3K pathway (INPP4B, PTEN). It was possible to identify a selective group of phosphatases upregulated only in the ER^−^ ERBB2^+^ subgroup and not in ER^+^ (like DUSP6, DUSP10 and PPAPDC1A among others), suggesting a role of these phosphatases in specific BC subtypes, unlike other differentially expressed phosphatases (DUSP4 and ENPP1) that seemed to have a role in multiple BC subtypes. Significant correlation was found at the protein level by IHC between the expression of DUSP6 and phospho-ERK (p=0.04) but not of phospho-ERK with DUSP4. To show the potential prognostic relevance of phosphatases as a functional group of genes, we derived and validated in two large independent BC microarray series a multiphosphatase signature enriched in differentially expressed phosphatases, to predict distant metastasis-free survival (DMFS). ER^−^ ERBB2^+^, ER^−^ ERBB2^−^ and ER^+^ BC patients have a distinct pattern of phosphatase RNA expression with a potential prognostic relevance. Further studies of the most relevant phosphatases found in this study are warranted.

## Introduction

Protein phosphatases are a diverse group of proteins that have in common the ability to dephosphorylate different substrates, predominantly proteins. Phosphatases have been recently classified in three major groups: the classic serine/ threonine (Ser/Thr) phosphatases, the protein tyrosine phosphatases (PTP), and the aspartate-based protein phosphatases (recently reviewed in refs. 1 and 2). This classification is based on the amino acid sequence of the catalytic domain and the structural similarity of these proteins. There are ~147 protein phosphatases in the human genome (1) and they participate in a number of critical biological processes such as proliferation, tumor suppression and motility. In the cells, a delicate balance is kept between protein kinases and phosphatases for the control of a variety of biological functions.

We previously found that the expression of the mitogen activated protein kinase-phosphatase 1 (MKP-1, also called DUSP1 or CL100), a dual specificity phosphatase whose known substrates are ERK, JNK and p38, is an independent prognostic factor in non-small cell lung cancer (NSCLC) patients, suggesting a potential role of this phosphatase in lung cancer (3). We have also previously shown that DUSP1 is differentially expressed in epithelial ovarian cancer as compared with normal ovarian epithelium. High levels of DUSP1 are found in normal ovarian epithelium whereas patients with advanced epithelial cancer tend to show a marked decrease in its expression. Induced reexpression of DUSP1 in ovarian cancer cell lines decreases their anchorage-dependent and -independent growth, indicating a potential role of this phosphatase in ovarian cancer progression (4).

Here, we wanted to explore the phosphatase transcriptome in different phenotypes of breast cancer (BC) patients with a particular focus in estrogen receptor-negative (ER^−^) BC patients by using expression microarrays. We characterize the ribonucleic acid (RNA) expression of phosphatases in estrogen receptor-positive (ER^+^), estrogen receptor-negative (ER^−^) BC and in the two major subgroups of ER^−^ BC [epidermal growth factor receptor 2-positive (ERBB2^+^) and epidermal growth factor receptor 2-negative (ERBB2^−^)] by expression microarrays. The potential relevance of both the MAPK pathway and the phosphoinositide-3-kinase (PI3K) pathways is inferred from the distinct phosphatase expression pattern in the ER^−^ BCs. Finally we also show the prognostic relevance of RNA expression of phosphatases in BC by building and validating a multiphosphatase signature predicting distant methastasis-free survival (DMFS) in untreated, lymph node-negative BC patients.

## Materials and methods

### Samples and patients

Forty-one fresh frozen samples corresponding to surgical specimens from BC primary tumors were used for the genomic study. Part of the tissue obtained at surgery was used for routine pathological evaluation of the samples, which also included immunohistochemistry (IHC) to assess estrogen receptor (ER), progesterone receptor (PGR) and ERBB2, and the rest was snap-frozen in OCT at −80°C shortly after surgery and stored in tumor banks at the two participating institutions. For the expression microarray study, samples were obtained from the tumor banks of Complejo Hospitalario Universitario of Santiago (35 samples) and from Hospital Quiron Torrevieja (6 samples) both in Spain. For the IHC study, formalin-fixed paraffin-embedded samples from primary BC were obtained from archival material at the Pathology department in Hospital Quiron Torrevieja (45 samples). All the samples were collected retrospectively following institutional review board approved protocols (i.e., approved by the respective ethics committees) at both institutions. Written informed consent prior to testing and publishing was obtained from all patients involved in the study.

Only samples that were ER^−^ by IHC were selected for this study. A perfect agreement was found with the microarray study as none of these samples expressed levels of ESR1 mRNA significantly above background level. All patients were also progesterone receptor-negative (PGR^−^) except one (from the expression microarray study), in which some expression of PGR was detected by IHC. As this tumor did not express any PGR in the microarray, it was considered as PGR^−^ for all the microarray analysis performed. Tumors were considered ERBB2^+^ if they had an HercepTest 3+, or had HercepTest 2+ and amplification of ERBB2 as shown by fluorescent *in situ* hybridization.

The samples studied in the microarray study contained ≥50% proportion of tumor tissue as verified by hematoxylin and eosin staining of one section of the frozen tissue taken prior to the collection of the sections used for total RNA extraction. All the clinical and pathological characteristics of the patients were extracted from the pathology reports. This study was approved by the Ethics Committee of Hospital Quiron Torrevieja, where the study was carried out.

### RNA handling and microarray processing

Total RNA extraction was done with RNAeasy columns (Qiagen, Hilden, Germany), and the amount obtained was measured with a Nanodrop espectophotometer (ND-1000, NanoDrop Technologies, Wilmington, USA). Quality of the RNA was measured with Agilent 2100 Bioanalyzer (Agilent Technologies, Waldbronn, Germany).

The oligonucleotide microarrays used for the 41 samples were the Whole Human Genome Microarray kit (4×44K) (Agilent Technologies, Palo Alto, CA, USA). The amount of total tumor RNA used for labeling was ~300 ng for the first 10 processed samples, and 200 ng for the remaining 31 samples. Tumor total RNA for all samples was labelled with Cy3 using the QuickAmp labeling kit, and the hybridisation kit (both from Agilent Technologies) according to the manufacturer’s recommendations. Two protocols were used: for the first 10 microarrays, a one-color protocol, and for the remaining 31 microarrays, a two-color protocol. As explained above, all tumor RNA samples were labelled with Cy3. For the two-color protocol used with the last 31 microarrays, in addition to the 200 ng of tumor RNA labelled with Cy3, labeling of a common reference RNA consisting of 200 ng of Universal Human reference RNA (Stratagene, CA, USA) with Cy5 was also performed (using also the QuickAmp labeling and hybridization kits from Agilent Technologies). Hibridization of the microarrays was done in a hybridization oven at 65°C for 17 h. All the microarrays hybridized were then scanned in a G2505B microarray scanner (Agilent Technologies). The raw data were extracted with Agilent Feature Extraction (version 9.5.1) software, and several quality control (QC) metrics (specifically up to 12 different metrics mainly related to the intensity and background of the spike-in control signals in the two channels) were applied according to the manufacturer’s recommendations. All the 41 microarrays were within acceptable ranges.

### Statistical analysis

For the analysis the R statistical environment (version 2.10.1) was used (http://cran.r-project.org/) along with packages from the BioConductor project (http://www.bioconductor.org/). As described above there were two groups of arrays: 10 hybridized according to one-color protocol, and 31 according to a two-color protocol. To make the two groups comparable, and to be able to analyse them jointly, avoiding any batch effects, the normalized signal (derived from Cy3, green channel) was chosen as a measurement of the signal intensity in both groups of arrays. Functions of the limma package (5) from the Bioconductor project were used for further preprocessing, that consisted of: background correction (normexp), quantile normalization among all the microarrays for interarray normalization and log2 transformation. QC filtering of probes was done by filtering out probes that were not expressed significantly above background levels in order to increase the signal to noise ratio. This filtering and summarization of identical probes repeated throughout the chip was done using the Bioconductor package Agi4×44PreProcess.

By using the green normalized signal the ranges of signal and background intensities were fully comparable between the one-color and the two-color microarrays as demonstrated by box plots. To further rule out any possible batch effect after preprocessing the 41 microarrays as mentioned above, unsupervised hierarchical clustering was performed. The 10 one-color microarrays did not form a separate cluster but rather mixed well with the remaining 31 arrays, ruling out in this way a batch effect.

The raw and preprocessed data from the 41 microarrays of the ER^−^ BC patients of this study have been deposited in the Gene Expression Omnibus repository (GEO accession no. GSE51999). For the different comparisons between two classes in BC patients described in Results statistical analysis of microarrays (SAM) was performed using the t-statistics of the siggenes package (from the Bioconductor project) with default parameters at the false discovery rate (FDR) indicated for each comparison. Each comparison was done selecting the probes representing many of the known phosphatase (and subunits) genes from the Bioconductor libraries corresponding to the chips analysed (Agilent hgug4112a and the Affymetrix hgu133a) in the different datasets used. The screening carried out in this study included all the probes containing the word ‘phosphatase’ in the description field of each chip library. A full list of the actual phosphatases screened (and their corresponding probes) is available from the authors upon request.

As explained in Results, the following published datasets were downloaded from the public domain: a) available from the GEO repository (all contain Affymetrix hgu133a microarrays): GSE7390 (198 patients) (6), GSE20194 (230 patients) (7), GSE2034 (286 patients) (8), and b) from http://microarray-pubs.stanford.edu/wound_NKI/explore.html (the microarrays correspond to an Agilent platform using a two-color protocol): the series published by Van de Vijver *et al* (295 patients) (9). All these series contain quality microarrays as selected by the authors of the respective publications (see the above publications for details). The preprocessing and summarization at the probe level of the Affymetrix hgu133a chips was done when possible from the original CEL files using the Robust Multiarray Average (RMA) algorithm as implemented in the Bioconductor affy package. If CEL files were not available, then the processed data were used as provided by the authors. For the Agilent arrays from the van Vijver *et al* (9) series the processed log ratios data (that are log10 transformed) were used as provided by the authors without further modification or filtering. The probes in the Affymetrix microarrays were annotated using the corresponding Bioconductor library. The Agilent microarrays processed log ratios were loaded into BRBArrayTools v2.7.0, software was designed by Amy Peng Lam and Richard Simon from the Biometric Research Branch Division of Cancer Treatment and Diagnosis of the National Cancer Institute (USA), and data were annotated through the Stanford SOURCE database.

For the inference of potential causative signaling pathways involved in the differential expression of phosphatases the Signaling Pathway Enrichment using Experimental Datasets (SPEED) web site was used (10) with default parameters.

For gene set enrichment analysis (GSEA) (11) Java GSEA desktop application software (version 2.0.13) was downloaded from the authors website (http://www.broadinstitute.org/gsea/downloads.jsp) along with the current MSigDB xml signatures file (version 4.0). Preranked GSEA was used with our ER^−^ BC series comparing ERBB2 enriched versus triple-negative (TN) or basal-like BC. All the preprocessed genes in the Agilent microarrays dataset were ranked using SAM analysis, and the results loaded in the software. The following parameters were used: 1,000 permutations, weighted enrichment statistics, exclusion of genesets with <15 genes and those with >500 genes, and the rest were the default.

For derivation of a multiphosphatase prognostic signature GSE2034 was used for training and GSE7390 for validation purposes (both use the Affymetrix hgu133a platform, include primary lymph node-negative patients, and contain distant metastases-free survival information). These two large series have been used extensively in the literature for survival analysis. Only the genes corresponding to all the phosphatases and subunits screened in this study were used (326 probes). To avoid any bias rather than selecting a subset of patients in each of these datasets, a whole dataset (GSE2034) was used for training, and then the signature was validated in the full GSE7390 dataset after performing z-score transformation of the 2 datasets. The derivation of this signature containing multiple phosphatases was based on a semisupervised approach (12) with some modifications. The multiphosphatase signature was derived from those phosphatases with the highest univariate Cox coefficients in GSE2034 according to a threshold of 1 (that was selected by cross-validation). Fifty-eight probes (corresponding to 48 genes) were selected for the signature.

Singular value decomposition of the gene expression matrix with the selected 58 features was carried out in the training set (GSE2034) to derive the scores of the principal components as follows:

(i) $$\text{V}={\text{X}}^{\text{T}}.\text{U}.{\text{D}}^{-1}$$

Here V is the principal component scores matrix, where for each column of V each row corresponds to a linear regression of the corresponding column of X. X is the p × n gene expression matrix with the selected 58 probes, where p are the features and n are the patients. U is an orthogonal matrix with the same number of columns as the transposed X (X^T^), selected so that the first columns of V represent the largest variance, and D is the diagonal matrix.

Then, the Cox proportional hazard regression model was fitted with the first 3 columns of V, representing the first 3 principal components to derive their coefficients. Finally, we use the Cox coefficients (β_v1_, β_v2_, β_v3_) obtained from the first 3 columns of V to derive an index score (I_j_) for each patient as a linear combination as follows:

(ii) $${\text{I}}_{\text{j}}=\beta_{\text{v}1}.{\text{V}}_{\text{j},1}+\beta_{\text{v}2}.{\text{V}}_{\text{j},2}+\beta_{\text{v}3}.{\text{V}}_{\text{j},3}$$

Where V_j,1_ is the V matrix values of the jth patient in the first column of V.

From this equation the higher the index scores (I_j_) the greater the risk of distant metastases. Likewise the V^test^ matrix of the principal component scores corresponding to the validation set (GSE7390) was calculated using the values of U and D^−1^ obtained from the training set in (i), with the transposed X^test^ matrix containing the expression values of the 58 selected probes of the multiphosphatase signature in GSE7390. Then, the signature index score for each patient of the validation set is obtained as in (ii) using the same coefficients calculated previously from the Cox proportional hazard regression model in the training set, but with the newly calculated V^test^ first 3 principal components scores from the validation set. Part of the first two steps was carried out using the R package superpc (for the obtention of the appropriate threshold and the selection of the phosphatases with the highest univariate Cox scores), and the last two steps with the R statistical environment.

Based on the value of the index score we could make separate groups of patients with prognostic significance in the training and validation datasets. Although statistically significant differences could be seen by using as cutoff the median of the score indexes (in the training dataset, log-rank p=0.0019) and almost significant (log-rank p=0.0658) in the validation dataset, the more pronounced and statistically significant differences in the DMFS were seen between the upper and lower quintiles of the signature score indexes. We found that a discrete group of patients with a strong statistically significant difference in DMFS could be made by comparing the three lower quintiles (of the value of the index scores) against the two upper quintiles (the ones with the highest index scores, in both the training and validation sets). To estimate the probability of the cumulative DMFS between the 2 groups of patients, Kaplan-Meier curves were drawn and the p-values between the two groups were obtained by log-rank test using SPSS (version 10.0).

For the multivariate analysis of the signature score indexes taken as both a continuous and a discrete variables (according to the separation of the 3 lower quintiles against the 2 upper quintiles, which was the optimal separation in 2 discrete groups in both the training and validation datasets), an approximation to obtain the hazard ratios was done by using the unstratified Cox proportional hazard regression model including as covariates known prognostic factors in BC that were available in the datasets used. SPSS software (version 10.0) was used for this purpose.

### Immunohistochemistry

The antibodies used were the rabbit polyclonal antibodies specific against the dual phosphorylated form of ERK1/2 (Thr202/Tyr204) (#4370, Cell Signaling, Beverly, MA, USA) at a dilution of 1:200, the polyclonal DUSP4 (MKP-2) antibody (NBP1-19592, Novus Biologicals, Littleton, CO, USA) at a dilution of 1:100, and a goat polyclonal anti-DUSP6 antibody (MKP-3) (sc-8599, Santa Cruz Biotechnology Lab Inc., Santa Cruz, CA, USA) at a dilution of 1:100, in the last case the anti-goat IG (HRP) (NB710-H, Novus Biologicals) was used as a secondary antibody at a dilution of 1:400.

For all immunohistochemical assays, 1,5-μm sections were cut from paraffin-embedded, formalin-fixed breast cancer tissue, each case was collected on xylanized slides. Endogenous peroxidase activity was blocked using 3% hydrogen peroxide in methanol for 15 min. Epitope retrieval was heat-induced in citrate buffer pH 6.0 and samples were incubated with each primary antibody at 4°C overnight. In the case of DUSP6, the secondary antibody was incubated at room temperature for one hour. Immunocytochemical reaction was shown using the EnVision™ intensifying kit (Dako, Carpinteria, CA, USA).

Commercially available slides with IHC controls (#8103, SignalSlide Phospho-p44/42 MAPK (Thr202/Tyr204) IHC Controls, Cell Signaling Technology) that consisted of paraffin-embedded NIH/3T3 cells, treated with U0126 (a specific and potent inhibitor of MEK1/2) or TPA (12-O-tetra-decanoylphorbol-13 acetate, a strong inducer of ERK1/2 activity through PKC modulation) were used as negative and positive controls for phospho-ERK1/2 antibody, respectively. Normal breast tissue included in the surgical specimens was evaluated as positive control for DUSP4 and DUSP6 antibodies. Negative control specimens in the absence of the primary antibodies DUSP4 and DUSP6, confirmed the specificity of the breast epithelial immunoreaction for these antibodies.

Immunoreactivity of the three antibodies was scored blindly in tissue sections identified only by the surgical accession number by two of the authors. Adequacy of IHC technique, was judged by the presence and intensity of immunoreaction in normal positive internal controls (normal breast epithelium) and the positive and negative controls of treated NIH/3T2 cells for the phospho-ERK1/2 antibody. The intensity of both cytoplasmic and nuclear staining detected by IHC was scored following a semiquantitative approach on a scale of 0–3+ (negative, 0; low-intensity positive staining, 1; moderate-intensity, 2; strong intensity, 3). The percentage of tumour cells demonstrating staining (either nuclear, cytoplasmic or both) was estimated for each sample. A categorical IHC classification was performed using a 4-tiered scale from 0 to 3; 0, no tumour cells stained or <5% of tumour cells demonstrating staining; 1, >5–33%; 2, 34–66%; and 3, >66% of tumour cells. The percentage of cells stained was used for the categorical groups created as explained below. The overall IHC score in each case was obtained as the product of the staining intensity and the actual percentage of cells and was used as a continuous measurement to assess correlations between the stainings of the 3 different antibodies. Each case was scored twice, independently by two of the authors, obtaining a good agreement with subsequent reconciliation of scored values.

For the IHC categorical data analysis the samples were divided for each antibody used in two categories, each containing ~50% of the tumors (for DUSP6 and phospho-ERK1/2), and almost 30 and 70% for DUSP4. The cutoff was <5% cells (i.e., 0) stained vs. the rest (scores 1–3) for DUSP6 and phospho-ERK1/2; and 0 and 1 IHC categorical score vs. 2–4 for DUSP4. Correlation of these groups with the tumor type (ER^−^ ERBB2^+^ or TN) was made by Fisher’s exact test. Spearman’s rho correlation coefficient was calculated for the pairwise comparison combinations of the three antibodies using the continuous score generated by the product of the intensity score by the percentage score.

### Co-expression network visualization

The GeneMANIA (version 3.1.2) plugin for Cytoscape (version 3.0.2) was used for phosphatase co-expression network visualization (based on Pearson pairwise correlation coefficients) using the public human data downloaded from the GeneMANIA server (13–15). The networks were explored with the desktop application but the representative figure was obtained from the server.

## Results and Discussion

### Microarray molecular profiling of the phosphatase transcriptome in estrogen receptor-negative breast cancer: clinical ERBB2 and triple-negative tumors

We studied the expression of 207 phosphatases and subunits (304 probes) by microarray profiling in a group of 41 primary BC patients with ER^−^ tumors. The characteristics of the patients presented here are shown in Table I. We compared in our series of ER^−^ BC, those ERBB2-overexpressing tumors (as determined by IHC), that we designated the clinical ERBB2, with the TN by using SAM analysis at a 5% FDR (q<0.05). Thirty-eight probes corresponding to 34 different genes were identified (Table II). The top phosphatases characterizing the clinical ERBB2 tumors that showed an ~1.5-fold change (or more) were DUSP6, DUSP4, FBP1, PPAPDC1A, ENPP1, INPP4B, PPAPDC1B, PTPRH, DUSP10, PPAPDC3, CTDSPL, PTEN and DOLPP1. The eight phosphatases identified showed an ~1.5-fold change (or more) difference in TN tumors: PPM1K, PTPLB, PSPH, PTPN14, PTPRE, PTPLA, PTPN2 and PPP1R12A. Given the important cellular functions of phosphatases, that keep a delicate balance in the phosphorilation status of different molecules, particularly kinases, we did not expect to find large fold changes in the comparisons made, as these changes would likely have important metabolic consequences.

Only one of the three series used to establish the most characteristic phosphatases in ER^+^ vs. ER^−^ BC (see below), provided information regarding the ERBB2 status of patients as determined by IHC: GSE20194. Therefore, we used the ER^−^ BC patients (n= 89) of the aforementioned series as a first validation of our results. SAM analysis at a 5% FDR (q<0.05) was also applied to this subgroup of patients comparing the clinical ERBB2 of this series with the TN tumors. Twenty-nine different probes were identified (Table II) corresponding to 20 different phosphatase genes. A total of 9 genes found in our series were also differentially expressed in the GSE20194 series of ER^−^ patients. However, several of the phosphatases found differentially expressed in our series were not present in the Affymetrix platform used in GSE20194.

### The phosphatome of ER^−^ BC patients in the two major molecular subgroups: ERBB2-enriched and basal-like enriched subtypes

**Figure 1:**
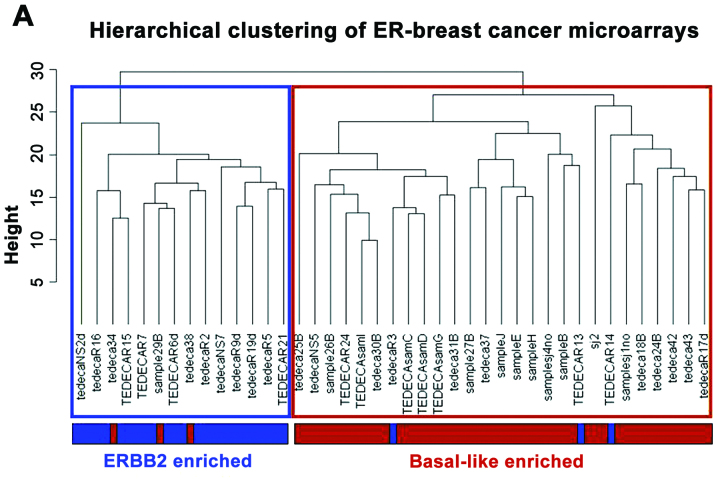

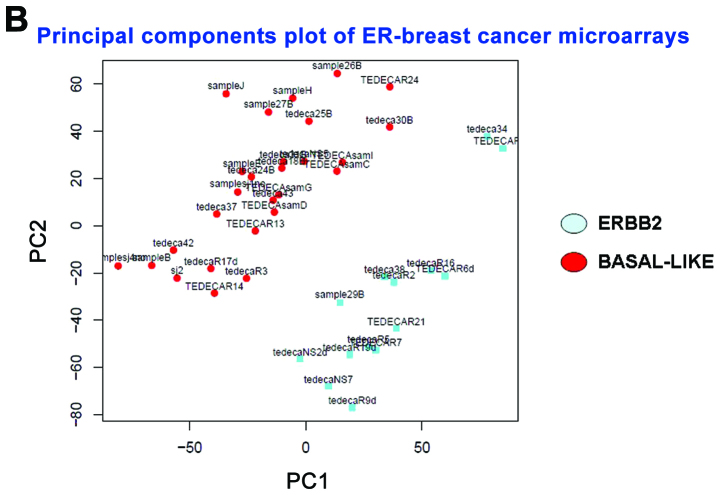
(A) Unsupervised hierarchical clustering (using euclidean distance metric and complete linkage) of the 41 samples from our ER^−^ BC patients, showing the ERBB2-enriched and the basal-like enriched clusters. (B) Plot of the first 2 principal components in our ER^−^ BC microarrays series.

Since the seminal study by Perou *et al* (16) describing the different molecular BC subtypes by using expression micro-arrays, it was noted that hierarchical clustering of ER^−^ tumors with the intrinsic signature genes yielded at least two clusters, one of them enriched in ERBB2 overexpressing tumors and another comprising mainly basal-like tumors. Although we applied a single sample predictor to the samples of our series using the classifier PAM50 published by Parker *et al* (17), with the exception of the basal-like subtype, the rest of the molecular subtypes did not have sufficient number of cases to analyze them separately (data not shown). Thus, we decided to apply hierarchical clustering to our samples using all the probes that in our microarray platform matched the intrinsic signature as reported by Hu *et al* (18). As expected, and noted previously by Perou and *et al* (16), mainly two clusters could be readily identified: the first was enriched in clinical ERBB2, containing 11 out of 14 (78%) of the clinical ERBB2 tumors (Fig. 1A), constituting the ERBB2-enriched molecular subtype; the second cluster contained the basal-like enriched tumors comprising TN (24 patients) and 3 clinical ERBB2 tumors. We designated the tumors belonging to the ERBB2 enriched cluster as molecular ERBB2 to distinguish them from the clinical ERBB2 (those tumors overexpressing ERBB2 by IHC), and the cluster of the predominant TN tumors as the basal-like enriched tumors even though there is a significant overlap among the clinical and molecular subtypes. We also used all the genes in the microarray to plot the first two principal components, showing that the same two groups characterized by hierarchical clustering can also be observed with a different unsupervised analysis (Fig. 1B). Given the reproducibility of this molecular classification of ER^−^ tumors we used it to validate further our results as we did not find other large breast cancer microarray series providing information regarding the ERBB2 status as determined by IHC. SAM analysis was also applied at a 5% FDR (q<0.05) to our series to identify differentially expressed phosphatases between the molecular ERBB2 and the basal-like enriched tumors. Forty-one probes (corresponding to 38 genes) were differentially expressed (Table III). Comparing with the phosphatases identified earlier in the comparison of clinical ERBB2 with TN tumors, 23 out of 41 (56%) probes (corresponding to 20 out of 38 genes) were common with the previous analysis (Tables II and III).

To further validate these results in another independent series, we used the NKI series (9) (295 patients) comprising 69 ER^−^ tumors. First, we applied hierarchical clustering to these 69 patients and as expected two major clusters could be identified, one comprising mainly the molecular ERBB2 and the other comprising the basal-like tumors. Then we applied SAM analysis at a 1% FDR (q≤0.01) to find genes differentially expressed between the molecular ERBB2 and the basal-like tumors. Twenty-two probes (corresponding to 21 genes) were differentially expressed (Table III). Eleven of these phosphatases were common with those found in our series.

### ER^+^ and ER^−^ BC patients also have a distinctive pattern of phosphatase expression

After analysing our series of estrogen receptor-negative (ER^−^) breast cancers we characterized the phosphatase transcriptome in the whole population of breast cancer patients encompassing both estrogen receptor-positive (ER^+^) and ER^−^ patients as a reference point to our study by using three large independent published microarrays series. Our purpose was to identify the phosphatases that were more characteristic of the major ER^−^ subgroups by taking into account the phosphatome of ER^+^ and ER^−^ BC phenotypes as a whole.

In order to identify phosphatases differentially expressed in ER^+^ versus ER^−^ breast cancer patients, we selected three large microarray series of breast cancer patients with the following characteristics: a) having ~200 or more patients so as to have statistical power, b) all performed in the same microarray platform to make possible a direct comparison of the same probes across the 3 series, c) all series must include information regarding the estrogen receptor status, and d) the series used do not have overlapping patients. The three series selected meeting these characteristics were downloaded from the public domain available at the GEO repository: GSE7390 (198 patients), GSE20194 (230 patients) and GSE2034 (286 patients). In all, the three series comprised 714 patients, and all used the Affymetrix HGU133A platform.

Each individual series was analyzed for differential expression of phosphatases between patients with ER^+^ vs. ER^−^ by SAM at a 5% FDR (q≤0.05). Out of a total of 326 probes (corresponding to 196 phosphatase and subunits genes) screened in the three series, 136 probes comprising 92 different genes were identified as differentially expressed in GSE7390, 144 probes (104 genes) in GSE20194, and 149 probes (106 genes) in GSE2034.

A total of 79 probes (Table IV) were identified as differentially expressed in each and every one of the three studied series. These 79 probes correspond to 62 different genes that can be consistently identified in the three series. All these 62 phosphatases were differentially expressed in the same manner in each series (i.e., the same phosphatases were predominantly expressed in either ER^+^ or in ER^−^ tumors in all series), as shown in Table IV. It is remarkable and interesting to note that out of 196 studied phosphatases 62 (31.6%, i.e., almost a third) were found differentially expressed by SAM at a stringent 5% FDR, suggesting that these genes may contribute in a relevant manner to the estrogen receptor driven phenotype of breast cancer.

In summary, pooling together the ER^−^ comparisons made between the two major subgroups, three phosphatases (DUSP4, DUSP6 and DUSP10) were consistently identified in our ER^−^ series (for both comparisons the clinical ERBB2 vs. TN and the molecular ERBB2 vs. the basal-like enriched tumors) and in the two independent series used for validation purposes, and 9 additional phosphatases (PPAPDC1A, DOLPP1, PTPN14, FBP1, ENPP1, INPP5A, LPPR2, PPP2R4 and PTPLA) were identified in our ER^−^ series (for both comparisons) and in at least one of the ER^−^ series used for validation. We consider that those phosphatases found in both our clinical ERBB2 vs. TN and in our molecular ERBB2 vs. basal-like enriched comparisons are likely to be the most relevant phosphatases of these ER^−^ subtypes. It is interesting to note that three of these phosphatases are dual specificity phosphatases (DUSP4, DUSP6 and DUSP10) and DUSP4 and DUSP6 share the same substrate: ERK (DUSP4 in addition to ERK also targets JNK and p38 kinases), suggesting that the control of the MAPK pathway through these phosphatases could be highly relevant to the biology of this subgroup of BC patients (ER^−^ ERBB2^+^). Another interesting observation related to these findings is that DUSP6, DUSP10, PPAPDC1A, DOLPP1 and INPP5A are phosphatases that we have identified upregulated (at ~1.5-fold or more) in the subgroup of ER^−^ that overexpress ERBB2 (or are enriched in ERBB2 over-expressing tumors). However, these genes were not picked as differentially expressed when comparing the phosphatases differentially expressed between ER^+^ and ER^−^ (Table IV) in the three large series analyzed in this study. This fact suggests that ER^−^ ERBB2^+^ BC patients tend to have upregulated some specific phosphatases that may be important for this subtype. However, DUSP4, FBP1, ENPP1, LPPR2 and PPP2R4 are upregulated in both ER^−^ ERBB2^+^ patients and in ER^+^ BC patients, whereas PTPN14 and PTPLA are upregulated in TN (and basal-like) and in all the ER^−^ BC patients, suggesting that these phosphatases also play a role in other BC subtypes.

As we have pointed out, not all the phosphatases screened in our platform and found differentially expressed in the comparisons made in our ER^−^ series, are actually represented in the other platforms used for validation purposes. Therefore, those differentially expressed phosphatases not represented in the other platforms might still be a true positive finding. Review of the literature of the phosphatases found differentially expressed in BC provided another source of validation for some of our findings, even for some that were not identified in the other two series used for validation. Two examples can be mentioned in this regard. Inositol polyphosphate 4-phosphatase type II (encoded by the gene INPP4B), a phosphatase that affects PI3K signaling by hydrolysis mainly of phosphatidyl inositol 3,4-biphosphate (PIP2) was found differentially overexpressed in ER^−^ ERBB2^+^ as compared with ER^−^ ERBB2^−^ tumors in our series of ER^−^ patients. It was also found overexpressed in ER^+^ BC patients as compared with ER^−^ patients in the screening carried out in this study. Gewinner *et al* found that the majority of TN BC tumors they studied had loss of heterozygosity at the 4q31.21 locus (where INPP4B resides), and that the messenger RNA expression of INPP4B was lower in this subgroup of BC patients (19). Further they also reported that decreased protein expression of INPP4B (as determined by IHC) correlated with a worse overall survival, suggesting that INPP4B behaves as a tumor suppressor (19). Fedele *et al* confirmed some of these findings and showed that indeed INPP4B protein is expressed at high levels in the normal breast, and predominantly in ER^+^ BC patients (20). PTEN was also identified as overexpressed in ER^−^ ERBB2^+^ in comparison with ER^−^ ERBB2^−^ in our series. Several previous reports have validated this finding at the protein level (21–23).

**Figure 2:**
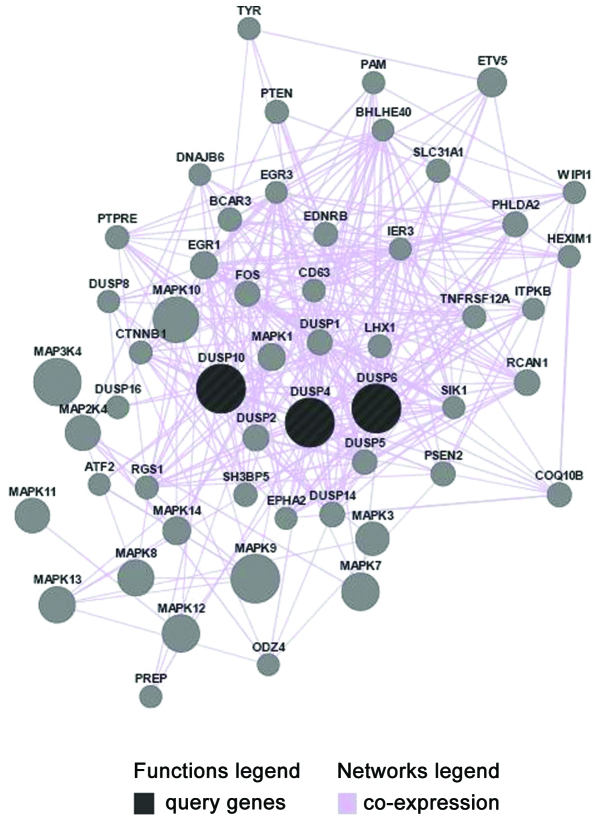
Co-expression network analysis from the GeneMania server using DUSP4, DUSP6 and DUSP10 as query genes.

Finally, we attempted to obtain insight into the function of the main phosphatases found differentially expressed between the two major ER-BC subgroups in all the series studied here including our own (i.e., DUSP4, DUSP6 and DUSP10) by using the GeneMANIA plugin for cytoscape in different human tumor datasets (Co-expression network in Fig. 2). Interestingly in two previous reports (24,25) a coexpression network, based on correlation coefficients, could be identified involving not only other MAPK phosphatases (like DUSP1, DUSP2 and DUSP5 among others) but also PTEN, suggesting a complex and intertwined regulation of phosphatases controlling the MAPK and PI3K pathways. Remarkably another phosphatase was part of the co-expression networks with DUSP4, DUSP6 and DUSP10: PTPRE. This phosphatase has been found to induce a positive feedback on ERK1/2 and AKT protein pathways in human breast cancer cells (26). Taken together, these data point to an important and complex regulatory function of different phosphatases in the control of the MAPK and PI3K pathways in BC.

### In silico inference of pathways involved in the differential regulation of phosphatase expression through gene expression patterns

As stated above, several upregulated phosphatases (DUSP4 and DUSP6) in ER^−^ ERBB2^+^ patients share ERK as a substrate, and others like INPP4B and PTEN regulate the PI3K pathway, so we investigated whether specific signaling pathways were likely to cause the regulation of expression shown above. For this purpose, we applied the Speed algorithm (10) to the top differentially expressed genes (not just the phosphatases) that were upregulated between clinical ERBB2 and TN tumors in our series as identified by SAM at a 1% FDR (q≤0.01) using in the comparison all the genes in our platform after QC filtering. The pathways that were significant (p<0.05) after adjustment for FDR are shown in Table V. Only three pathways were significant out of 9 considered: the MAPK_only (adjusted p=2.01e^−7^), the MAPK_PI3K (p=0.0423) and the transforming growth factor β (TGF-β) pathway (adjusted p=0.0036). As suspected by the phosphatases having ERK as substrate, it seems that one of the major signaling pathways driving their regulation is the MAPK pathway with a contribution from the PI3K pathway.

In a similar manner, we also run the Speed algorithm with the top genes that were upregulated in TN (and therefore downregulated in clinical ERBB2), as picked by SAM at a 1% FDR. Six pathways (out of 9) were significant (Table V) at an adjusted p<0.05: the MAPK_only, MAPK_PI3K, interleukin-1 (IL1), toll-like receptor (TLR), tumor necrosis factor α (TNFα) and the Wnt signaling pathways, being the Wnt and the TLR pathways the most significant of all (Wnt adj p=4.577e^−7^ and TLR adj p=3.975e^−7^). When running the Speed algorithm in a similar way on the top genes upregulated in molecular ERBB2 and in the basal-like tumors of our series, similar results were obtained (Table V). The most significant pathway was the MAPK pathway (adj p=3.5117e^−16^) in the molecular ERBB2. In the basal-like tumors three pathways were the most significant: Wnt pathway (adj p=2.62e^−5^), IL1 (adj p=2.38e^−5^) and TLR (adj p=2.49e^−5^). However, MAPK (adj p=0.02158) and PI3K (adj p=0.047) pathways were also significant in the basal-like subgroup of ER^−^ BC, suggesting a role for these pathways in the expression of some of the phosphatases studied here.

Trying to confirm a potential role for the pathways studied above with a different statistical approach we also performed preranked GSEA analysis of our ER^−^ BC series. Analysis was carried out with the Broad Institute collection of signatures MsigDB (version 4.0) as explained in Materials and methods, and we focused on the most significant hits obtained from the C6 geneset collection corresponding to oncogenic signatures. In Table VI the three most significant hits are shown for the four categories of patients (molecular ERBB2, basal-like, clinical ERBB2 and TN). Both the clinical and molecular ERBB2 had as highly significant hits the activated ERBB2 and MEK1 signatures (FDR q-value ≤0.01 for both). The ERBB2 and MEK1 signatures were generated in a human ER^+^ breast cancer cell line (MCF-7) overexpressing constitutively activated ERBB2 or activated MEK1 (the upstream ERK1/2 kinase), respectively, suggesting a potential role of the MAPK pathway in the ERBB2-enriched patients, and possibly in the regulation of the expression of those phosphatases having ERK as substrate. In addition, these 2 subgroups (clinical and molecular ERBB2) had in common another significant hit (FDR≤0.01): a KRAS.PROSTATE_UP.V1_UP signature obtained in epithelial prostate cancer cell lines over-expressing an oncogenic form of KRAS, suggesting a role for both the MAPK and PI3K pathways in the regulation of expression of the phosphatases detected in ER^−^ ERBB2^+^ BCs. Exploring signatures overlapping with the activated MEK and the KRAS.PROSTATE_UP.V1_UP signatures, a signature of *ETS2* regulated gene targets was found to be a highly significant overlap (FDR q-value = 5.03e^−11^ and 6.44e^−3^, respectively). It is known that *DUSP6* has an ETS2 site in its promoter that *in vitro* is responsive to MAPK activation (reviewed in ref. 27). However, it has not been shown in ER^−^ BC tissues whether there is a close correlation between the activation of the MAPK pathway and the protein expression of DUSP6.

### IHC of DUSP4, DUSP6 and phospho-ERK1/2 in ER^−^ BC tissues

We decided then to study at the protein level by IHC the expressions of DUSP4, DUSP6 and its relationship with the activated form of ERK (as detected by a phospho-ERK1/2 specific antibody) in an independent series of 45 ER^−^ BC patients (12 ER^−^ ERBB2^+^, 33 TN), and to focus on the clinical classification of BC tumors for this IHC study as similar pathway analysis results were found with the molecular ER^−^ classification (see above).

As expected, we found differences in protein expression of DUSP6 and phospho-ERK between the clinical ER^−^ subgroups (ERBB2^+^ and TN) but not for DUSP4, following the same trend observed for the RNA expression in the microarray analysis (i.e., higher expression of DUSP6 and phospho-ERK in ER^−^ ERBB2^+^ than in TN), although these differences were not statistically significant considering a categorical classification of the expression of these proteins (Fisher’s exact test p-values (two sided) p=0.176 for DUSP6 and p=0.179 for phosho-ERK), likely due to the limited number of ER^−^ ERBB2^+^ tumors analyzed (Table VII).

By using a continuous IHC score of the expression of these proteins we found a statistically significant correlation [Spearman’s rho = 0.307, p-value (2-tailed) = 0.043] between phospho-ERK and DUSP6, but not between phospho-ERK and DUSP4 [Spearman’s rho = 0.136, p-value (2-tailed) = 0.379]. There was also a significant correlation between DUSP6 and DUSP4 [Spearman’s rho = 0.415, p-value (2-tailed)= 0.005]. These results in ER^−^ BC tissues suggest first that the protein expression of DUSP6 is linked to the activation of ERK1/2 and second that the expression of DUSP4 depends not only on the activation of ERK1/2 but also on other factors, as this phosphatase has other substrates in addition to ERK1/2 (i.e., JNK and p38). Some transcription factors known to be substrates of these kinases are also part of co-expression networks (like FOS for MAPK and ATF2 for p38 pathways) as shown above.

### Association between prognosis and phosphatase RNA expression

**Figure 3:**
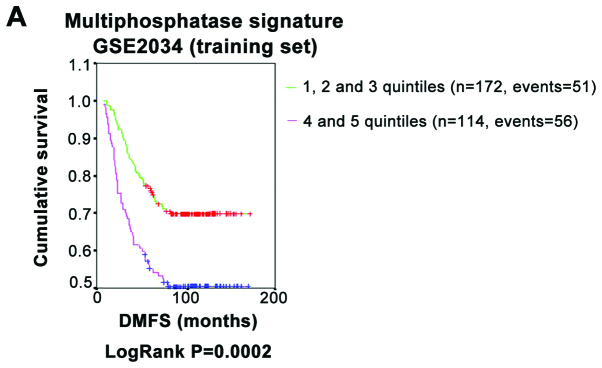

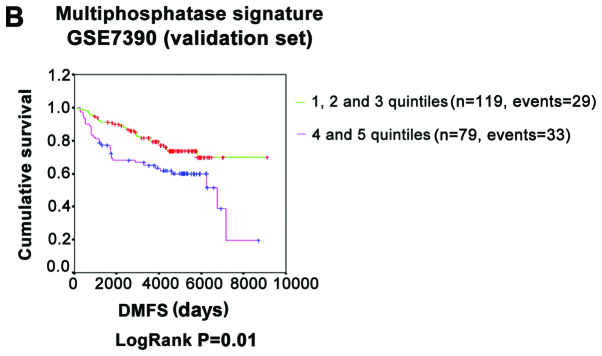
(A) Kaplan-Meier plot of 2 prognostic groups obtained according to the 58 probes (48 genes) multiphosphatase signature trained in GSE2034 and (B) tested in GSE7390.

To study whether the differential pattern of expression of phosphatases studied above had not just a relationship with the BC phenotype but also a potential association with prognosis, we focused on two of the series we used for comparison between ER^+^ and ER^−^ tumors (GSE2034 and GSE7390) as the third one used (GSE20194) did not provide survival information. These two series included information on DMFS that was used for this analysis. In addition, both series included untreated, lymph node-negative BC patients. Hence these two large series were ideal to explore a potential association between the distant metastases-free survival and the expression of the phosphatases screened in our study. Using as a starting point all the phosphatases screened here, we were able to find a multigene signature in the whole population of BC patients (considering both ER^+^ and ER^−^ patients) with a highly statistically significant prognostic value. We used as training set all the BC patients in the GSE2034 dataset in order to obtain a 58 probes signature (comprising 48 genes) (Table VIII) and we validated this signature in the GSE7390 dataset, that was used as the validation set (HR=2.718, 95% CI=1.616–4.571; p<0.001 for the training set and HR=3.005, 95% CI=1.315–6.870; p=0.009 for the validation set when the signature was used as a continuous variable). Fig. 3, shows the Kaplan-Meier curves of the 2 datasets using the optimal cutoff of the signature score, i.e., the 3 lower quintiles versus the 2 upper quintiles (log-rank test p=0.0002 for the training set and p=0.01 for the validation set). Using this signature in the GSE7390 validation dataset (that provided more information about known clinical prognostic factors) as a continuous variable, it was found to retain statistical significance in predicting DMFS in a multivariate Cox proportional hazard regression model adjusted for other known prognostic factors (HR=2.784, 95% CI=1.086–7.136, p=0.033) (Table IX). The same was true for the training dataset (GSE2034 series), although in this series there was a reduced amount of provided information on other known prognostic factors (data not shown). We also used the multiphosphatase signature as a discrete variable (with the optimal separation of 2 groups of patients corresponding to the 3 lowest quintiles and the 2 upper quintiles, respectively) in the GSE7390 validation dataset, and it was also found to retain statistical significance in a multivariate Cox regression model (following a backward elimination method based on the Wald test) along with tumor size [signature: HR=1.755, 95% CI=1.061–2.903, p=0.028, and tumor size (continuous): HR=1.394, 95% CI=1.049–1.852, p=0.022), whereas estrogen receptor status, age and grade (all as discrete variables) were not significant and were eliminated and not retained in the minimum optimal model. Similarly the signature as a discrete variable was also highly significant in the training set after adjusting for other potential prognostic factors (data not shown).

To further confirm the prognostic value of the 48 genes used in the multiphosphatase signature, as an independent confirmation, we used an online database where a simplified model of the signature used in our study is used as explained (28). In brief, the linear part of a multivariate Cox model is used by these authors to obtain a prognostic index, i.e., they use directly the Cox coefficients as weights of the expression of the genes used in the generation of their prognostic index. We could confirm utilizing all the available genes (and probes where applicable) of our multiphosphatase signature in the Aguirre-Gamboa *et al* (28) database that with exactly the same probes and genes used in our study a highly statistically significant prognostic model (with the same or analogous endpoint, DMFS or RFS) could be fit not only to the same BC datasets used to train and validate our signature, but also to other breast cancer datasets we tried (which were those with the larger number of patients) in this database [namely: GSE2990 (n=187), GSE6532 (n=214), GSE4922 (n=249), E-TABM-158 (n=117), GSE20685 (n=327), and finally a pool of 9 breast cancer datasets (n=676)] (data not shown]. These data suggest the robustness of these genes to predict DMFS and RFS in BC.

It is noteworthy that a number of phosphatases that were part of the signature were those that had been identified as differentially expressed in the previous analysis comparing ER^+^ vs. ER^−^ patients (like DUSP4, INPP5J, PTP4A2 and PPP2R2A) as well as others that had been identified in the ER^−^ ERBB2^+^ vs. ER^−^ ERBB2^−^ analysis (like DUSP6).

In this study we characterized the differential expression of phosphatases that accompany the most relevant phenotypic subtypes of BC by gene expression profiling using microarrays, with a particular focus on ER^−^ BC. Although there is a previous molecular profiling study by microarrays of the tyrosine phosphatome of ERBB2 overexpressing BC by Lucci *et al* (29), a different procedure was used. In the study of Lucci *et al* only the protein tyrosine phosphatases were studied with a custom microarray in breast cancer cell lines under different conditions. Then Lucci *et al* also studied two different BC datasets where they compared ERBB2^+^ vs. ERBB2^−^ in the whole population of BC patients (i.e., including both ER^+^ and ER^−^ tumors). Thus they did not separate them according to their ER status. Nevertheless, in common with our study, they identified DUSP6 and DUSP10 as differentially expressed between ERBB2^+^ and ERBB2^−^, being DUSP6 the most significant finding (29).

To the best of our knowledge our study represents the first thorough characterization of the transcriptome of most of the known phosphatases in BC phenotypes according to their ER status in 3 large independent microarrays series. Here, ER^+^ BC tumors could be considered as a surrogate of the luminal subtype. Our study also provides a characterization of the phosphatome of the 2 major molecular subgroups of ER^−^ tumors: ERBB2 overexpressing and ERBB2^−^ (basal-like). In order to achieve this in the ER^−^ subgroup, we used the data generated by our own series of ER^−^ BC patients and validated our findings in at least 2 large independent microarrays series. Further validation of some of our findings was provided by a literature review as stated earlier for PTEN and INPP4B (19–23).

Estrogen regulation may explain other expression changes observed in our comparison of ER^+^ vs. ER^−^ phosphatases. PTPN13 (also known as PTPL1) was found overexpressed in ER^+^ patients. A previous report showed a positive statistically significant correlation between the expression of this phosphatase as measured by quantitative real-time PCR and hormonal receptor status in BC patients, thus confirming our observation (30).

Recently, a study of predictive biomarkers of efficacy of trametinib (GSK1120212), a new inhibitor of MEK1/2 (2 kinases that are upstream of ERK1/2 in the MAPK pathway) that is being tested in clinical trials (31), has shown in multiple human cancer cell lines that the RNA expression of DUSP6 is associated with sensitivity to this compound irrespective of the mutational status of RAS/RAF, thus behaving as a surrogate marker of MAPK activation, and as a predictor of sensitivity to MEK inhibitors. Our study supports the association between the expression of DUSP6 and the activation of ERK1/2 at the protein level in ER^−^ BC, suggesting that DUSP6 could be used in these patients as a predictive biomarker for treatment with MEK inhibitors, like trametinib.

The pathway analysis carried out in this study in ER^−^ BCs, derived from the differential expression of phosphatases, lends support to other reports in the literature of BC regarding the role of the MAPK (32) and PI3K pathways in ER^−^ BCs in both ERBB2^+^ and ERBB2^−^ patients (20,23,33). However, in addition, it supports that multiple phosphatases targeting the MAPK and PI3K pathways act in a coordinated manner to control the regulation of these pathways as shown by the co-expression network analysis included in this study, suggesting cross-talk at different levels of the two pathways mediated, at least in part, by different phosphatases. A recent report by Will *et al* (34) further supports these observations. In BC cell lines with amplified ERBB2, inhibitors of PI3K pathway are effective in causing apoptosis, that is dependent on a transient inhibition of ERK activation, suggesting that it could be of clinical relevance in these subgroups of BC patients to inhibit both pathways as shown by Will *et al* (34). It is also of interest that the report of Will *et al* corroborates previous reports placing the RAS-ERK pathway downstream of PI3K under certain cellular contexts.

We were able to generate and validate in two large independent BC microarrays series (comprising 486 patients) a multiphosphatase signature in untreated, lymph node-negative primary BC patients (both ER^+^ and ER^−^) with highly statistically significant differences in DMFS. Our purpose was only to show the potential prognostic relevance of phosphatases as a functional group of genes. It is noteworthy that a significant number of the phosphatases comprising the signature were found differentially expressed in this study. The signature found would need further validation to consider it in the clinical setting, but as pointed out, it was not our purpose, as we did not choose other genes, different from phosphatases or their subunits to generate the signature, that could certainly be more strongly correlated with DMFS in the GEO studies analyzed. It is interesting to note that phosphatases such as DUSP4 and PTPRC, that are in our signature, are actually part of published BC prognostic signatures (8,35). Lower levels of DUSP4 are associated with worse prognosis in our multi-phosphatase signature, and also in a recent report profiling residual BCs after neoadjuvant chemotherapy (36).

In conclusion, we characterized the distinctive phosphatome of the major BC phenotypes (ER^−^ ERBB2^+^, ER^−^ ERBB2^−^, ER^+^), and provide evidence of the relevance of the MAPK and PI3K pathways in ER^−^ BC as potential drivers of several of the differentially expressed phosphatases. The findings suggest that these pathways might be of potential therapeutic interest in these patients. We also show that the expression of DUSP6 could be used as a surrogate marker of MAPK activation, and hence as a potential predictive biomarker of activity of MAPK pathway inhibitors in ER^−^ BCs. Finally, we show the prognostic value of coordinated phosphatase RNA expression in primary BC by generating and validating a multiphosphatase signature enriched in differentially expressed phosphatases.

## Figures and Tables

**Table I:** Characteristics of the 41 ER^−^ BC patients investigated in the microarray study.

Age, median	62 (range 34–80)
Diameter (mm)	25 (range 14–75)
ER	
Negative	41/41 (100%)
PGR	
Negative	40/41 (97.6%)
Positive	1/41 (2.4%)
ERBB2	
Negative	26/41 (63.4%)
Positive^a^	14/41 (34.2%)
NA	1/41 (2.4%)
Grade	
G1	1/41 (2.4%)
G2	13/41 (31.7%)
G3	23/41 (56.1%)
NA	4/41 (9.8%)
pT	
T1c	9/41 (22%)
T2	28/41 (68.3%)
T3	3/41 (7.3%)
T4a	1/41 (2.4%)
pN	
0	24/41 (58.5%)
1	7/41 (17.1%)
2	4/41 (9.8%)
3	5/41 (12.2%)
NA	1/41 (2.4%)
Lymph nodes	
0	24/41 (58.5%)
1	6/41 (14.6%)
≥3	10/41 (24.4%)
NA	1/41 (2.4%)
Histology	
DIC	35/41 (85.4%)
Medular	3/41 (7.3%)
Metaplastic	3/41 (7.3%)

a All ERBB2^+^ tumors are Herceptest 3+.

**Table II:** Phosphatases differentially expressed in clinical ER^−^ ERBB2^+^ versus triple-negative (TN) BC patients in this series (Agilent platform) and in GSE20194 (Affymetrix platform) (both FDR q-value <0.05).

Agilent platform			Affymetrix platform		
	
Probe ID	Symbol	Fold change	Probe ID	Symbol	Fold change
A_23_P139704	**DUSP6**	2.37430542	204014_at	**DUSP4**	3.72
A_23_P134935	**DUSP4**	2.21815412	204015_s_at	**DUSP4**	2.42
A_23_P257111	**FBP1**	2.21456226	208892_s_at	**DUSP6**	2.28
A_23_P156880	**ENPP1**	2.15344204	208891_at	**DUSP6**	1.98
A_24_P810290	**PPAPDC1A**	2.04366557	208893_s_at	**DUSP6**	1.98
A_23_P18559	INPP4B	1.9332961	209696_at	**FBP1**	1.61
A_23_P322845	PPAPDC1B	1.84368063	209457_at	DUSP5	1.58
A_23_P101642	**PTPRH**	1.80689287	218273_s_at	PDP1	1.55
A_23_P51856	**DUSP10**	1.63243567	221563_at	**DUSP10**	1.51
A_24_P182494	**DUSP10**	1.58973789	205066_s_at	**ENPP1**	1.44
A_23_P157736	PPAPDC3	1.55666487	201702_s_at	PPP1R10	1.38
A_24_P251534	CTDSPL	1.52956909	203006_at	**INPP5A**	1.38
A_24_P913115	PTEN	1.4922811	201703_s_at	PPP1R10	1.36
A_23_P386764	DOLPP1	1.49053177	203997_at	PTPN3	1.3
A_23_P81880	CTDSP2	1.48543867	217844_at	CTDSP1	1.3
A_24_P26897	**INPP5A**	1.47424739	215501_s_at	**DUSP10**	1.28
A_23_P60458	**PPP2R4**	1.43930243	204555_s_at	PPP1R3D	1.27
A_23_P15348	MPRIP	1.43095284	213651_at	INPP5J	1.26
A_23_P394014	MPRIP	1.42031077	204554_at	PPP1R3D	1.25
A_23_P153461	**LPPR2**	1.41127758	201598_s_at	INPPL1	1.23
A_23_P125505	PPEF1	1.39364806	208300_at	**PTPRH**	1.23
A_23_P35796	PPP2R5B	1.37299248	206452_x_at	**PPP2R4**	1.22
A_23_P53390	PTPRB	1.34635634	218509_at	**LPPR2**	1.22
A_24_P279328	INPP5K	1.3263309	216105_x_at	**PPP2R4**	1.21
A_23_P385017	G6PC	1.30644385	208874_x_at	**PPP2R4**	1.2
A_23_P154771	DUSP15	0.74429683	204578_at	HISPPD2A	1.19
A_24_P63109	PPP1R2	0.71850718	64899_at	**LPPR2**	1.15
A_24_P4705	PPME1	0.69793884	213368_x_at	PPFIA3	1.14
A_32_P1445	PTPN2	0.66623065	201409_s_at	PPP1CB	0.69
A_24_P364412	PPP1R12A	0.66335654			
A_23_P207940	PTPN2	0.65350836			
A_24_P213503	PTPRE	0.6457558			
A_23_P161352	PTPLA	0.62997551			
A_24_P213494	PTPRE	0.62600872			
A_24_P2648	PTPN14	0.59729493			
A_23_P251987	PSPH	0.58411377			
A_23_P155197	PTPLB	0.52918763			
A_24_P214598	PPM1K	0.439163			

Phosphatases in bold are those in common between our series and GSE20194.

**Table III:** Phosphatases differentially expressed between the molecular ER^−^ ERBB2^+^ and the basal-like enriched BC in this series (FDR q-value <0.05) and in the NKI series (FDR q-value ≤0.01).

Agilent platform (hgug4112a)			Agilent platform (NKI dataset)		
	
Probe ID	Symbol	Fold change	UG cluster	Symbol	Fold change
A_23_P139704	**DUSP6**	4.120910839	Hs.417962	**DUSP4**	2.2438
A_23_P257111	FBP1	3.569938845	Hs.75431	ALPL	2.0417
A_23_P134935	**DUSP4**	3.213163487	Hs.298654	**DUSP6**	1.6904
A_23_P156880	ENPP1	3.111709348	Hs.2128	**DUSP5**	1.5135
A_23_P18559	INPP4B	2.890365054	Hs.40479	**PPAPDC1A**	1.4791
A_24_P810290	**PPAPDC1A**	2.269061201	Hs.177534	**DUSP10**	1.4554
A_23_P35414	PPP1R3C	1.970501973	Hs.435238	PPP1R1A	1.3803
A_23_P302494	PPP1R3D	1.937966763	Hs.21701	**DOLPP1**	1.3489
A_23_P110712	DUSP1	1.910555827	Hs.74624	PTPRN2	1.3369
A_23_P123336	PDP1	1.852692665	Hs.409834	**PHPT1**	1.2717
A_23_P157736	PPAPDC3	1.79895655	Hs.444468	**CTDSP1**	1.2022
A_23_P51856	**DUSP10**	1.755823276	Hs.444468	**CTDSP1**	1.2022
A_23_P150018	**DUSP5**	1.735497207	Hs.156814	HISPPD2A	1.1901
A_24_P182494	**DUSP10**	1.698858704	Hs.13854	PPTC7	0.7943
A_23_P201808	PPAP2B	1.676793454	Hs.143137	NANP	0.7943
A_23_P125505	PPEF1	1.568005282	Hs.512667	**PTPN14**	0.7762
A_23_P83192	**PHPT1**	1.531733681	NA	PTPNS1	0.7762
A_23_P53390	PTPRB	1.52381411	Hs.181236	MTMR2	0.7413
A_23_P151297	TENC1	1.515222957	Hs.78867	PTPRZ1	0.6918
A_23_P81880	CTDSP2	1.512888492	Hs.114062	**PTPLA**	0.5248
A_23_P60458	PPP2R4	1.498501858	Hs.5753	**IMPA2**	0.5012
A_23_P18493	PTPN13	1.487651286	Hs.144879	DUSP9	0.1954
A_23_P153461	LPPR2	1.482240041			
A_23_P156667	PPP1R10	1.478707737			
A_23_P75299	LHPP	1.477347827			
A_23_P28263	**CTDSP1**	1.470771458			
A_24_P26897	INPP5A	1.468964472			
A_24_P913115	PTEN	1.46501226			
A_23_P35796	PPP2R5B	1.432704786			
A_23_P386764	**DOLPP1**	1.426831074			
A_23_P111240	PHACTR2	1.266553393			
A_23_P347048	SGPP1	1.223263923			
A_23_P89762	PHLPP1	0.771400312			
A_23_P149111	**PTPN14**	0.700547814			
A_23_P163143	ACYP1	0.687477537			
A_32_P1445	PTPN2	0.656101811			
A_23_P207940	PTPN2	0.628126761			
A_23_P420692	PPFIA4	0.593573284			
A_23_P161352	**PTPLA**	0.564620489			
A_24_P2648	**PTPN14**	0.503665086			
A_23_P50081	**IMPA2**	0.376755182			

Phosphatases in bold are those found to be common between our series and the NKI series.

**Table IV:** Phosphatases differentially expressed between ER^+^ and ER^−^ BC in common among GSE7390, GSE20194 and GSE2034 (FDR q-value ≤0.05).

Probe ID	Symbol	Up in
208617_s_at	PTP4A2	ER^+^
209696_at	FBP1	ER^+^
208616_s_at	PTP4A2	ER^+^
216988_s_at	PTP4A2	ER^+^
208615_s_at	PTP4A2	ER^+^
44654_at	G6PC3	ER^+^
205948_at	PTPRT	ER^+^
221759_at	G6PC3	ER^+^
208652_at	PPP2CA	ER^+^
204284_at	PPP1R3C	ER^+^
217844_at	CTDSP1	ER^+^
213651_at	INPP5J	ER^+^
218540_at	THTPA	ER^+^
202432_at	PPP3CB	ER^+^
205066_s_at	ENPP1	ER^+^
204014_at	DUSP4	ER^+^
201906_s_at	CTDSPL	ER^+^
204015_s_at	DUSP4	ER^+^
212494_at	TENC1	ER^+^
204578_at	HISPPD2A	ER+
203445_s_at	CTDSP2	ER+
213795_s_at	PTPRA	ER^+^
205376_at	INPP4B	ER^+^
203029_s_at	PTPRN2	ER^+^
204201_s_at	PTPN13	ER^+^
202313_at	PPP2R2A	ER^+^
203966_s_at	PPM1A	ER^+^
202187_s_at	PPP2R5A	ER^+^
213521_at	PTPN18	ER^+^
209457_at	DUSP5	ER^+^
216105_x_at	PPP2R4	ER^+^
217777_s_at	PTPLAD1	ER^+^
213799_s_at	PTPRA	ER^+^
206452_x_at	PPP2R4	ER^+^
218509_at	LPPR2	ER^+^
201904_s_at	CTDSPL	ER^+^
218961_s_at	PNKP	ER^+^
204160_s_at	ENPP4	ER^+^
208874_x_at	PPP2R4	ER^+^
203030_s_at	PTPRN2	ER^+^
212686_at	PPM1H	ER^+^
209585_s_at	MINPP1	ER^+^
204161_s_at	ENPP4	ER^+^
209817_at	PPP3CB	ER^+^
202165_at	PPP1R2	ER^+^
203126_at	IMPA2	ER^−^
212680_x_at	PPP1R14B	ER^−^
207749_s_at	PPP2R3A	ER^−^
201407_s_at	PPP1CB	ER^−^
203038_at	PTPRK	ER^−^
200913_at	PPM1G	ER^−^
213136_at	PTPN2	ER^−^
204207_s_at	RNGTT	ER^−^
219654_at	PTPLA	ER^−^
204852_s_at	PTPN7	ER^−^
209632_at	PPP2R3A	ER^−^
205194_at	PSPH	ER^−^
212640_at	PTPLB	ER^−^
209633_at	PPP2R3A	ER^−^
202883_s_at	PPP2R1B	ER^−^
200637_s_at	PTPRF	ER^−^
201409_s_at	PPP1CB	ER^−^
218845_at	DUSP22	ER^−^
213137_s_at	PTPN2	ER^−^
220236_at	PDPR	ER^−^
204208_at	RNGTT	ER^−^
204553_x_at	INPP4A	ER^−^
201629_s_at	ACP1	ER^−^
204049_s_at	PHACTR2	ER^−^
206060_s_at	PTPN22	ER^−^
204048_s_at	PHACTR2	ER^−^
204469_at	PTPRZ1	ER−
205503_at	PTPN14	ER−
204507_s_at	PPP3R1	ER−
214771_x_at	MPRIP	ER^−^
212197_x_at	MPRIP	ER^−^
41577_at	PPP1R16B	ER^−^
202513_s_at	PPP2R5D	ER^−^
215227_x_at	ACP1	ER^−^

**Table V:** Adjusted p-values (with FDR correction) after applying the Speed algorithm (based on Fisher’s exact test) to the clinical and molecular classifications of ER^−^ BC of our series as explained in the text.

	Clinical and molecular subgroups of ER− BC patients			
	
Pathways	Clinical ERBB2	Triple-negative	Molecular ERBB2	Basal-like enriched
MAPK_only	2.0177e-07	0.0010214	3.5117e-16	0.02158
MAPK_PI3K	0.042333	0.0061918	2.778e-05	NS
PI3K_only	NS	NS	NS	0.047341
TGF-β	0.0036155	NS	8.5908e-09	NS
TLR	NS	3.975e-07	NS	2.4919e-05
TNFα	NS	0.0010083	2.9701e-06	0.00045425
IL1	NS	8.5305e-07	0.023904	2.3885e-05
Wnt	NS	4.577e-07	NS	2.6202e-05

NS, not significant (adjusted p-value >0.05). VEGF pathway has also been explored but it was NS for the 4 subgroups.

**Table VI:** Most significant results of GSEA analysis with oncogenic signatures.

Name	ES	NES	NOM p-val	FDR q-val
A) Molecular ERBB2				
ERB2_UP.V1_UP	0.511	2.278	0.000	0.000
MEK_UP.V1_UP	0.484	2.197	0.000	0.000
KRAS.PROSTATE_UP.V1_UP	0.551	2.109	0.000	0.001
B) Basal-like				
RPS14_DN.V1_DN	−0.396	−1.967	0.000	0.003
CSR_LATE_UP.V1_UP	−0.382	−1.889	0.000	0.005
GCNP_SHH_UP_EARLY.V1_UP	−0.388	−1.886	0.000	0.005
C) Clinical ERBB2				
ERB2_UP.V1_UP	0.431	1.921	0.000	0.009
KRAS.PROSTATE_UP.V1_UP	0.493	1.872	0.001	0.014
MEK_UP.V1_UP	0.418	1.870	0.000	0.014
D) Triple-negatives				
ERB2_UP.V1_DN	−0.429	−2.186	0.000	0.000
HINATA_NFKB_TARGETS_KERATINOCYTE_UP	−0.427	−2.010	0.000	0.003
HINATA_NFKB_TARGETS_FIBROBLAST_UP	−0.423	−1.961	0.000	0.004

ES, enrichment score. NES, normalized enrichment score. NOM p-val, nominative probability value. FDR q-val, false discovery rate q-value.

**Table VII:** DUSP4, DUSP6 and phospo-ERK1/2 immuohistochemical percentage scores within the triple-negative breast carcinomas and the ERBB2-positive, ER and PR-negative breast carcinoma groups.

Percentage of expression score	Triple-negative BC No. of cases/total (%)	ER^−^ ERBB2^+^ No. of cases (%)	p-value (two tailed, Fisher’s exact test)
DUSP6			
0	20/33 (60.6)	4/12 (33.3)	0.176
1–3	13/33 (39.4)	8/12 (66.7)	
DUSP4			
0–1	9/32 (28.1)	3/12 (25)	1.0
2–3	23/32 (71.9)	9/12 (75)	
P-ERK1/2			
0	19/32 (59.4)	4/12 (33.3)	0.179
1–3	13/32 (40.6)	8/12 (66.7)	

DUSP4 and P-ERK1/2 have one missing data each because all the material available was exhausted for one of the samples.

**Table VIII:** Multiphosphatase signature comprising 58 probes (48 genes) trained in GSE2034 training set and validated in GSE7390 (both Affymetrix HGU133A platform).

Probe ID	Symbol	Raw score	Differentially expressed
204014_at	DUSP4	−2.24	Yes
204015_s_at	DUSP4	−2.652	Yes
212587_s_at	PTPRC	−1.684	
209392_at	ENPP2	−1.432	
207238_s_at	PTPRC	−1.14	
204960_at	PTPRCAP	−1.27	
210839_s_at	ENPP2	−1.76	
208893_s_at	DUSP6	−1.079	Yes
203332_s_at	INPP5D	−1.337	
41577_at	PPP1R16B	−1.036	Yes
213651_at	INPP5J	−1.506	Yes
201904_s_at	CTDSPL	−1.466	Yes
216988_s_at	PTP4A2	−1.562	Yes
204852_s_at	PTPN7	−1.477	Yes
200637_s_at	PTPRF	−1.601	Yes
212750_at	PPP1R16B	−1.06	Yes
209457_at	DUSP5	−1.634	Yes
211178_s_at	PSTPIP1	−1.208	
200635_s_at	PTPRF	−1.42	Yes
203011_at	IMPA1	1.876	
200695_at	PPP2R1A	−1.304	
202313_at	PPP2R2A	−1.047	Yes
218852_at	PPP2R3C	1.233	
209896_s_at	PTPN11	−1.014	
212494_at	TENC1	−1.275	Yes
203253_s_at	HISPPD1	1.184	
204048_s_at	PHACTR2	1.031	Yes
212230_at	PPAP2B	−1.113	Yes
204566_at	PPM1D	1.91	
201603_at	PPP1R12A	1.205	Yes
207000_s_at	PPP3CC	−1.239	
206547_s_at	PPEF1	1.454	Yes
212640_at	PTPLB	1.538	Yes
209895_at	PTPN11	−1.238	
204554_at	PPP1R3D	1.724	Yes
203555_at	PTPN18	−1.052	Yes
219235_s_at	PHACTR4	−1.218	
201598_s_at	INPPL1	1.124	Yes
218516_s_at	IMPAD1	1.343	Yes
202429_s_at	PPP3CA	1.517	
202794_at	INPP1	−1.064	
200726_at	PPP1CC	1.579	
202425_x_at	PPP3CA	1.442	
218576_s_at	DUSP12	1.314	
206833_s_at	ACYP2	−1.057	
202066_at	PPFIA1	2.244	
214978_s_at	PPFIA4	1.368	
203338_at	PPP2R5E	1.097	
217956_s_at	ENOPH1	2.002	
200733_s_at	PTP4A1	1.564	Yes
206844_at	FBP2	−1.144	
212610_at	PTPN11	1.396	
202464_s_at	PFKFB3	2.534	
215066_at	PTPRF	1.301	Yes
203607_at	INPP5F	1.355	
201702_s_at	PPP1R10	1	Yes
214043_at	PTPRD	1.478	
202457_s_at	PPP3CA	1.956	

Raw score represents the univariate Cox coefficients for each gene of the signature. Overall, when overexpressed, genes with negative raw scores are associated with good prognosis, and when overexpressed, genes with a positive raw score are associated with poor prognosis.

**Table IX:** Multivariate Cox hazard regression model in GSE7390 (validation set) with the multiphosphatase signature as a continuous variable adjusted for known potential prognostic factors.

	Hazard ratio	95% confidence interval	p-value
Age (<50 vs ≥50)	0.795	(0.462–1.367)	0.407
Size	1.426	(1.068–1.906)	0.016
Grade (1 and 2 vs 3)	1.687	(0.868–3.280)	0.123
ER (− vs +)	1.850	(0.986–3.470)	0.055
Signature	2.784	(1.086–7.136)	0.033

## Abbreviations

BC: breast cancer
DMFS: distant metastasis-free survival
ER: estrogen receptor
ER^−^: estrogen receptor-negative
ER^+^: estrogen receptor-positive
ERBB2^+^: epidermal growth factor receptor 2-positive
ERBB2^−^: epidemal growth factor receptor 2-negative
FDR: false discovery rate
ERK: extracellular signal-regulated kinase
IHC: inmunohistochemistry
HRP: horseradish peroxidase
MAPK: mitogen-activated protein kinase
PGR: progesteron receptor
PGR^−^: progesterone receptor-negative
PI3K: phosphoinositide-3-kinase
RNA: ribonucleic acid
SAM: statistical analysis of microarrays
TLR: toll-like receptor
IL1: interleukin-1
TGF-β: transforming growth factor β
TNFα: tumor necrosis factor α
pcs: principal components
GEO: Gene Expression Omnibus
